# Epidemiology, vaccine effectiveness, and risk factors for mortality for pneumococcal disease among hospitalised adults in Singapore: a case-control study

**DOI:** 10.1186/s12879-020-05140-1

**Published:** 2020-06-17

**Authors:** Tyson Chan, Min Zhi Tay, Win Mar Kyaw, Angela Chow, Hanley J. Ho

**Affiliations:** 1 Department of Clinical Epidemiology, Office of Clinical Epidemiology, Analytics, and Knowledge, Tan Tock Seng Hospital, 11 Jalan Tan Tock Seng, Singapore, 308433 Singapore; 2 Preventive Medicine Residency Programme, National University Health System, Singapore, Singapore

**Keywords:** Pneumococcal infections, Pneumococcal vaccines, Epidemiology, Mortality, Singapore

## Abstract

**Background:**

*Streptococcus pneumoniae* infections can lead to severe morbidity and mortality, especially in patients with invasive pneumococcal disease (IPD). This study evaluated factors associated with pneumococcal disease, pneumococcal vaccine effectiveness, and risk factors for all-cause mortality in hospitalised adults with pneumococcal disease in Singapore.

**Methods:**

Retrospective case-control study of patients tested for pneumococcal disease with streptococcal urinary antigen testing and at least one sterile site culture, during their admission to a tertiary hospital in Singapore from 2015 to 2017. Patients were defined as cases of IPD or non-IPD, or as controls, based on laboratory results and clinical diagnoses. Multivariable models were constructed to determine factors associated with IPD/non-IPD, and risk factors for mortality from pneumococcal disease. Vaccine effectiveness against IPD/non-IPD was estimated using a variation of the test-negative design.

**Results:**

We identified 496 pneumococcal disease cases, of whom 92 (18.5%) had IPD. The mean age of cases was 69.1 ± 15.4 years, and 65.5% were male. Compared with controls (*N* = 9181), IPD patients were younger (mean age 61.5 ± 16.3 years, vs 72.2 ± 16.1 years in controls; *p* < 0.001) and with less co-morbidities [median Charlson’s score 1 (IQR 0–4), vs 3 (1–5) in controls; *p* < 0.001]. IPD patients also had the highest proportions with intensive care unit (ICU) admission (20.7%), inpatient mortality (26.1%) and longest median length of stay [9 (IQR 8–17) days]. On multivariable analysis, IPD was negatively associated with prior pneumococcal vaccination (adjusted relative risk ratio = 0.20, 95%CI 0.06–0.69; *p* = 0.011). Risk factors for mortality among pneumococcal disease patients were ICU admission, diagnosis of IPD, age ≥ 85 years and Charlson’s score > 3.

**Conclusion:**

Patients with pneumococcal disease (especially IPD) were younger and had less co-morbidities than controls, but had higher risk of severe clinical outcomes and mortality. Pneumococcal vaccination effectiveness against IPD was estimated to be about 80%, and should be encouraged among high-risk patients.

## Background

Pneumococcal disease is caused by *Streptococcus pneumoniae* and can lead to severe clinical outcomes and death*. S. pneumoniae* is the most common cause of community-acquired pneumonia (CAP), estimated to cause 27.3% of CAPs worldwide [1], and accounting for 29.2% of isolated pathogens from CAPs in Asia [2]. Other common sites of infection include otitis media and sinusitis. Invasive pneumococcal disease (IPD), defined as isolation of *S. pneumoniae* from a normally sterile site (such as blood or cerebrospinal fluid), carries an even higher risk of mortality and morbidity [3].

Cases of pneumococcal disease were predominantly male, and patients 65 years and above had the highest hospitalisation rates and case-fatality rates [4, 5]. From 1995 to 2004, the overall mean annual hospitalisation rate for pneumococcal disease in Singapore was 10.9 per 100,000 population and the case-fatality rate was 3.2% [4]. Specifically for IPD, locally reported case-fatality rates have ranged from 13.1 to 21.4% [6]_._

Transmission of the pneumococcus occurs via respiratory droplets from people with pneumococcal disease or asymptomatic individuals with carriage of the organism in their nasopharynx [7]. Risk factors for pneumococcal disease include presence of chronic diseases involving cardiovascular, pulmonary, hepatic, neurological or endocrine systems; immunosuppressed states; cigarette smoking history; alcohol abuse; recent influenza infection; institutionalised status; male sex and extremes of age [8]. Successful empiric antibiotic therapy is vital to avoid treatment failure and subsequent costs [9]. However, increasing antibiotic resistance in *S. pneumoniae* in the community has been reported [10]. Hence, while updated treatment recommendations (especially for CAP) have reduced disease-associated complications and mortality, the risks from infection still remain significant [11].

Adult vaccination is an effective measure for upstream prevention of pneumococcal disease. Both the 13-valent pneumococcal conjugate vaccine (PCV13) (which covers for serotypes 1, 3, 4, 5, 6A, 6B, 7F, 9 V, 14, 18C, 19A, 19F and 23F) and 23-valent pneumococcal polysaccharide vaccine (PPSV23) (which covers for serotypes 1, 2, 3, 4, 5, 6B, 7F, 8, 9 N, 9 V, 10A, 11A, 12F, 14, 15B, 17F, 18C, 19A, 19F, 20, 22F, 23F and 33F) [12], are effective against IPD [13–15]. PCV13 has also been shown to reduce the risk of subtype-specific non-invasive pneumococcal pneumonia [14]. Adult immunisation with both PCV13 and PPSV23 in elderly and high-risk groups is recommended by the United States Advisory Committee on Immunisation Practices (ACIP) [16]. The Singapore Ministry of Health (MOH) also recommended PPSV23 vaccination in adults in 2009 and subsequently both PCV13 and PPSV23 in 2017 [17]. However, pneumococcal vaccination uptake in adults has been low, estimated to be 6.1% among adults ≥65 years in 2013 [18].

Previous studies in Asia have described the changing epidemiology of pneumococcal disease due to introduction of pneumococcal vaccines [19, 20]. However, there have been few reports on the epidemiology of hospitalised adult patients with pneumococcal disease. Furthermore, evaluating the vaccine effectiveness in this group of patients may help strengthen the case for hospital-based and nationwide adult vaccination programmes.

The aims of this study were to evaluate the factors associated with pneumococcal disease, vaccine effectiveness in preventing severe outcomes of pneumococcal disease, and the risk factors for all-cause mortality in hospitalised adults with pneumococcal disease in Singapore.

## Methods

### Patient population and study design

We conducted a case-control study on patients suspected to have pneumococcal disease admitted to Tan Tock Seng Hospital (TTSH), the second largest adult acute tertiary care hospital in Singapore with 1600 beds, from January 2015 through December 2017. Our participants comprised all patients (aged 16 years and above) admitted over the study period who were evaluated for pneumococcal disease using streptococcal urinary antigen testing and at least one sterile site clinical culture during their admission. Cases of pneumococcal disease were defined as either having i) a positive result on testing for streptococcal urinary antigen, or ii) growth of *S. pneumoniae* from sterile site cultures. For patients with multiple admissions for pneumococcal disease, only the first admission was included for this study.

We further stratified cases into those with IPD versus those with non-IPD. IPD was defined as either having i) a sterile site culture (e.g. blood, joint fluid, or pleural fluid) positive for *S. pneumoniae,* or ii) a positive streptococcal urinary antigen test and a clinical diagnosis suggesting invasive disease (e.g. meningitis, septic arthritis, or pleural empyema), with cultures from the relevant site negative for any other causative pathogen. A non-IPD case was defined as a patient with a positive streptococcal urinary antigen test, but without positive cultures or any clinical diagnosis suggesting invasive disease.

To identify factors associated with IPD and non-IPD, the cases were compared to a control group comprising the remainder of the participants. For clarity, controls were defined as patients admitted to TTSH over the same study period, with a negative test for streptococcal urinary antigen, and all laboratory cultures yielding negative results for *S. pneumoniae*. Again, for patients with multiple admissions, only the first admission fulfilling the definition was considered. Patients who did not have both streptococcal urinary antigen tests and at least one sterile site culture (typically, at least one blood culture) performed during their admission were excluded from this study.

### Data sources and classification

All data for the study was captured using the hospital’s electronic medical record system, which comprehensively captures the demographics, clinical characteristics, laboratory results, and vaccination records of patients. Pneumococcal vaccination was defined as ever having any record for PCV13 and/or PPSV23 given at least 14 days prior to the date of hospital admission. Inpatient mortality was defined as death resulting from any cause during the pneumococcal disease-related admission. International Classification of Diseases, 10th Revision (ICD-10) was used to establish patient diagnoses, comorbidities, risk factors, and infectious disease history. The co-morbidity status was assessed according to Charlson’s co-morbidity score classification [21].

### Statistical analysis

Descriptive statistics were analysed for all study participants and for patient subgroups, including the number and proportions for categorical characteristics, mean and standard deviation for normally distributed continuous variables, and median and interquartile range (IQR) for non-normally distributed variables.

We subsequently analysed differences in patient characteristics across our three patient subgroups (IPD, non-IPD and controls). Differences in proportions were assessed using Chi-square or Fisher’s exact tests for categorical variables, while continuous variables were evaluated using the one-way ANOVA (for means) or Kruskal-Wallis test (for medians) as appropriate. Multinomial regression analysis was used to further evaluate patient factors associated with IPD and non-IPD, as IPD, non-IPD and non-pneumococcal disease patients were considered as three unranked categories of patients, based on their laboratory results and clinical diagnosis. This also enabled us to analyse our entire study cohort using a single model. Factors to include in the model were determined based on a *p* value threshold of 0.05 on univariate analysis, using non-pneumococcal disease patients as the base comparison group, with calculation of adjusted relative risk ratios (RRRs) for independent variables. Within our multivariable model, we included pneumococcal vaccination status (receipt of PCV13 and/or PPSV23 vaccination at least 14 days prior to admission date) as a variable to determine its effectiveness in reducing risk of IPD / non-IPD. Vaccination effectiveness was calculated using the formula [(1 - RRR) × 100%], instead of the usual [(1-odds ratio, OR) × 100%] used in the test-negative design. This was a variation of the test-negative design used for vaccine effectiveness studies, using multinomial logistic regression to construct the statistical model.

Among the cases, we also evaluated factors associated with inpatient all-cause mortality. Univariate analysis of demographic and clinical variables was first performed, and variables were included in a multiple logistic regression model again based on a *p* value threshold of 0.05 on univariate analysis, with adjustment for demographic confounders. Unadjusted and adjusted odds ratios (ORs) and 95% confidence intervals (CIs) were calculated to determine the strength of association between variables and in-hospital all-cause mortality.

For all analyses, a *p* value of < 0.05 was considered statistically significant. Furthermore, inclusion of variables into multivariable logistic regression models also took into account confidence intervals and clinical significance of variables. All statistical analyses were performed using Stata version 13 (StataCorp 2013, College Station, TX).

## Results

A total of 496 cases with pneumococcal disease were identified over the study period (Table 1). Cases were predominantly elderly (mean age 69.1 ± 15.4 years), male (*N* = 325, 65.5%), and of Chinese ethnicity (*N* = 359, 72.4%). The median Charlson’s comorbidity score was 2 (IQR 1–4). The most common co-morbidities were chronic pulmonary disease (30.0%), diabetes mellitus (29.6%), myocardial infarction/congestive cardiac failure (28.8%), and renal disease (18.8%). Majority had pneumonia as their primary diagnosis (70.0%). Eighty (16.1%) had received pneumococcal vaccination (PCV13 and/or PPSV23) at any time prior to admission. Ninety-two (18.5%) cases had IPD. Within this group, 84 (91.3%) had growth of *S. pneumoniae* in blood cultures only, 3 (3.3%) in pleural fluid cultures only, and 1 (1.1%) each in cerebrospinal fluid and joint fluid cultures only. A further 1 (1.1%) case had both blood and cerebrospinal fluid cultures positive, 1 (1.1%) had both blood and joint fluid cultures positive, and 1 (1.1%) was culture negative, but had a diagnosis of meningitis and a positive streptococcal urinary antigen test, with no other organisms implicated.

**Table 1:** Demographic and clinical characteristics and outcomes of hospitalised patients with pneumococcal disease

Characteristics	Pneumococcal disease (***N*** = 496), N(%)
Age, mean (years) ± SD	69.1 ± 15.4
*Age group*	
< 65 years	170 (34.3)
65-74 years	125 (25.2)
75-84 years	131 (26.4)
≥ 85 years	70 (14.1)
Gender = male	325 (65.5)
*Ethnicity*	
Chinese	359 (72.4)
Malay	61 (12.3)
Indian	39 (7.9)
Others	37 (7.5)
Nursing home residence	4 (0.8)
*Vaccination status (received ≥ 14 days prior to admission)*	
PCV13	6 (1.2)
PPSV23	78 (15.7)
Any pneumococcal vaccination	80 (16.1)
Influenza vaccination in past 1 yr	75 (15.1)
*Co-morbidities*	
Charlson’s score, median (IQR)	2 (1–4)
Charlson’s score > 3	137 (27.6)
Myocardial infarction/Congestive heart failure	143 (28.8)
Cerebrovascular disease	66 (13.3)
Dementia	54 (10.9)
Chronic pulmonary disease	149 (30.0)
Liver disease	27 (5.4)
Diabetes mellitus	147 (29.6)
Renal disease	93 (18.8)
Any malignancy	53 (10.7)
Hemiplegia	18 (3.6)
Peripheral vascular disease	22 (4.4)
Peptic ulcer disease	17 (3.4)
Connective tissue disease	8 (1.6)
AIDs	3 (0.6)
*Primary diagnosis*	
Pneumonia	347 (70.0)
Sepsis	63 (12.7)
Meningitis	5 (1.0)
Other respiratory diseases	73 (14.7)
Other diagnoses	8 (1.6)
*Outcomes*	
ICU admission	54 (11.0)
Length of stay (days), median (IQR)	8 (5–14)
Length of stay > 7 days	275 (55.4)
In-hospital mortality	70 (14.1)
30-day in-hospital mortality	33 (6.7)
*Readmission for any pneumococcal infection*	
Within 30 days from discharge date	1 (0.2)
Anytime during study period	6 (1.2)

*SD* standard deviation, *PCV13* 13-valent pneumococcal conjugate vaccination, *PPSV23* 23-valent pneumococcal polysaccharide vaccine, *IQR* inter-quartile range, *ICU* intensive care unit

When comparing IPD patients (*N* = 92), non-IPD patients (*N* = 404) and non-pneumococcal disease patients (*N* = 9181)(Table 2), significant differences across groups were observed for age (mean age in controls: 72.2 ± 16.1 years, non-IPD patients:70.8 ± 14.7 years, IPD patients: 61.5 ± 16.3 years; *p* < 0.001), gender (with higher proportions of males among IPD and non-IPD patients), history of pneumococcal vaccination (IPD patients: 3.3%, non-IPD patients: 19.1%, controls: 18.5%; *p* = 0.001), influenza vaccination in the past 1 yr before admission (IPD patients: 6.5%, non-IPD patients: 17.1%, controls: 15.2%; *p* = 0.039), and Charlson’s co-morbidity score (median score in controls: 3, non-IPD patients: 2, IPD patients: 1; *p* < 0.001). In terms of clinical outcomes, IPD was associated with the highest proportion of intensive care unit (ICU) admissions (20.7%, versus 8.7% in non-IPD patients and 6.3% in controls; *p* < 0.001) and in-hospital all-cause mortality (26.1% versus 11.4 and 9.1% respectively; p < 0.001). Similarly, length of stay was longest for IPD patients (median duration 9 days, versus 8 days in non-IPD and control groups; *p* = 0.003).

**Table 2:** Univariate and multinomial logistic regression analysis of demographic and clinical characteristics of hospitalised patients with invasive, non-invasive, and non-pneumococcal disease

Characteristics	IPD (N = 92), N(%)	Non-IPD (N = 404), N(%)	Non-pneumococcal disease (N = 9181), N(%)	p value for trend	RRR_**1**_ (95% CI) (IPD vs non-pneumococcal disease)	p value	RRR_**2**_ (95% CI) (Non-IPD vs non-pneumococcal disease)	p value
Age, mean (years) ± SD	61.5 ± 16.3	70.8 ± 14.7	72.2 ± 16.1	< 0.001				
*Age group*				< 0.001				
< 65 years	50 (54.4)	120 (29.7)	2449 (26.7)		Ref		Ref	
65-74 years	22 (23.9)	103 (25.5)	1885 (20.5)		0.65 (0.39–1.09)	0.101	1.17 (0.89–1.54)	0.258
75-84 years	14 (15.2)	117 (29.0)	2719 (29.6)		0.31 (0.17–0.56)	< 0.001	0.96 (0.74–1.25)	0.774
≥ 85 years	6 (6.5)	64 (15.8)	2128 (23.2)		0.19 (0.09–0.44)	< 0.001	0.72 (0.52–0.98)	0.038
Gender = male	66 (71.7)	259 (64.1)	5035 (54.8)	< 0.001	1.87 (1.18–2.96)	0.008	1.42 (1.15–1.76)	0.001
*Ethnicity*				0.202				
Chinese	63 (68.5)	296 (73.3)	6940 (75.6)					
Malay	11 (12.0)	50 (12.4)	852 (9.3)					
Indian	11 (12.0)	28 (6.9)	788 (8.6)					
Others	7 (7.6)	30 (7.4)	601 (6.6)					
Nursing home residence	2 (2.2)	2 (0.5)	96 (1.1)	0.247				
*Vaccination status (received ≥ 14 days prior to admission)*								
PCV13	1 (1.1)	5 (1.2)	105 (1.1)	0.861				
PPSV23	2 (2.2)	76 (18.8)	1632 (17.8)	< 0.001				
Any pneumococcal vaccination	3 (3.3)	77 (19.1)	1695 (18.5)	0.001	0.20 (0.06–0.69)	0.011	1.05 (0.77–1.43)	0.771
Influenza vaccination	6 (6.5)	69 (17.1)	1392 (15.2)	0.039	0.83 (0.34–2.02)	0.685	1.16 (0.84–1.60)	0.371
*Co-morbidities*								
Charlson’s score, median (IQR)	1 (0–4)	2 (1–4)	3 (1–5)	< 0.001				
Charlson’s score > 3	26 (28.3)	111 (27.5)	3753 (40.9)	< 0.001	0.72 (0.45–1.15)	0.170	0.55 (0.44–0.68)	< 0.001
Myocardial infarction / Congestive heart failure	31 (33.7)	112 (27.7)	2718 (29.6)	0.492				
Cerebrovascular disease	6 (6.5)	60 (14.9)	1737 (18.9)	0.001				
Dementia	3 (3.3)	51 (12.6)	1529 (16.7)	< 0.001				
Chronic pulmonary disease	14 (15.2)	135 (33.4)	2513 (27.4)	0.001				
Liver disease	5 (5.4)	22 (5.5)	604 (6.6)	0.667				
Diabetes mellitus	24 (26.1)	123 (30.5)	3548 (38.7)	< 0.001				
Renal disease	19 (20.7)	74 (18.3)	2665 (29.0)	< 0.001				
Any malignancy	11 (12.0)	42 (10.4)	1232 (13.4)	0.201				
*Outcomes*								
ICU admission	19 (20.7)	35 (8.7)	574 (6.3)	< 0.001				
Length of stay (days), median (IQR)	9 (8–17)	8 (5–14)	8 (5–13)	0.003				
Length of stay > 7 days	71 (77.2)	204 (50.5)	4918 (53.6)	< 0.001				
In-hospital mortality	24 (26.1)	46 (11.4)	836 (9.1)	< 0.001				
30-day in-hospital mortality	8 (8.7)	25 (6.2)	465 (5.1)	0.096				
*Readmission for any pneumococcal infection*								
Anytime during study period	0 (0.0)	6 (1.5)	16 (0.2)	0.001				
Within 30 days from discharge date	0 (0.0)	1 (0.25)	2 (0.02)	0.146				

Variables included in the final multivariable analysis were age group, gender, any pneumococcal vaccination, influenza vaccination, and Charlson’s score > 3. *IPD* invasive pneumococcal disease, *RRR* adjusted relative risk ratio, *CI* confidence interval, *SD* standard deviation, *PCV13* 13-valent pneumococcal conjugate vaccination, *PPSV23* 23-valent pneumococcal polysaccharide vaccine, *IQR* inter-quartile range, *ICU* intensive care unit

In our multivariable models (Table 2), IPD patients were much less likely than controls to have a history of any pneumococcal vaccination prior to admission (RRR = 0.20, 95%CI 0.06–0.69; *p* = 0.011). IPD was also positively associated with male gender (RRR = 1.87, 95%CI 1.18–2.96; *p* = 0.008), and negatively associated with increasing age groups, with very elderly patients (≥85 years) being less likely to have IPD compared to younger counterparts (< 65 years) (*p* < 0.001). Although influenza vaccination was negatively associated with IPD on univariate analysis, this factor was non-significant after multivariable adjustment. Non-IPD patients were also more likely than controls to be of male gender, although the effect size was smaller than in IPD patients (RRR = 1.42, 95%CI 1.15–1.76; *p* = 0.001). Patients ≥85 years and those with Charlson’s score of > 3 were also less likely to have non-IPD, and no significant differences in vaccination status was observed.

Of the 496 patients with pneumococcal disease, 70 (14.1%) died during their admission (Table 3). The case fatality rates for IPD and non-IPD were 26.1 and 11.4% respectively. Patients who died were more likely to be older (mean age 74.4 ± 14.3 years vs 68.2 ± 15.4 years; *p* < 0.001), have a Charlson’s score > 3, a diagnosis of IPD, or ICU admission. Presence of co-morbidities such as myocardial infarction/congestive heart failure, cerebrovascular disease and dementia were also significantly associated with mortality. After adjustments in our multivariable models, factors significantly and independently associated with mortality from pneumococcal disease were age ≥ 85 years (compared to below 65 years) (adjusted OR, AOR = 10.78, 95%CI 4.10–28.37; *p* < 0.001), Charlson’s score > 3 (AOR = 1.93, 95%CI 1.02–3.64; *p* = 0.042), diagnosis of IPD (AOR = 3.19, 95%CI 1.55–6.59; *p* = 0.002) and ICU admission (AOR = 23.22, 95%CI 11.07–48.70; p < 0.001).

**Table 3:** Univariate and multiple logistic regression analysis of demographic and clinical factors associated with all-cause mortality among pneumococcal disease patients

Characteristics	Died (***N*** = 70), N(%)	Survived (***N*** = 426), N(%)	OR (95%CI)	p value	AOR (95% CI)	p value
Age, mean (years) ± SD	74.4 ± 14.3	68.2 ± 15.4		< 0.001		
*Age group*						
< 65 years	16 (22.9)	154 (36.2)	Ref	Ref	Ref	Ref
65-74 years	18 (25.7)	107 (25.1)	1.62 (0.79–3.32)	0.188	1.70 (0.69–4.15)	0.246
75-84 years	16 (22.9)	115 (27.0)	1.34 (0.64–2.79)	0.435	1.99 (0.80–4.94)	0.138
≥ 85 years	20 (28.6)	50 (11.7)	3.85 (1.85–8.00)	< 0.001	10.78 (4.10–28.37)	< 0.001
Gender = male	45 (64.3)	280 (65.7)	0.94 (0.55–1.59)	0.814	1.05 (0.55–1.98)	0.892
*Ethnicity*						
Chinese	51 (72.9)	308 (72.3)	Ref	Ref		
Malay	9 (12.9)	52 (12.2)	1.05 (0.49–2.25)	0.910		
Indian	7 (10.0)	32 (7.5)	1.32 (0.55–3.15)	0.530		
Others	3 (4.3)	34 (8.0)	0.53 (0.16–1.80)	0.311		
*Vaccination status (received ≥ 14 days prior to admission)*						
Any pneumococcal vaccination	7 (10.0)	73 (17.1)	0.54 (0.24–1.22)	0.138		
Influenza vaccination in past 1 yr	8 (11.4)	67 (15.7)	0.69 (0.32–1.51)	0.354		
*Co-morbidities*						
Charlson’s score, median (IQR)	1 (0–4)	2 (1–4)		< 0.001		
Charlson’s score > 3	30 (42.7)	107 (25.1)	2.24 (1.33–3.77)	0.002	1.93 (1.02–3.64)	0.042
Myocardial infarction / Congestive heart failure	30 (42.9)	113 (26.5)	2.08 (1.24–3.49)	0.006		
Cerebrovascular disease	17 (24.3)	49 (11.5)	2.47 (1.33–4.60)	0.004		
Dementia	16 (22.9)	38 (8.9)	3.03 (1.58–5.79)	0.001		
Chronic pulmonary disease	16 (22.9)	133 (31.2)	0.65 (0.36–1.18)	0.160		
Liver disease	3 (4.3)	24 (5.6)	0.75 (0.22–2.56)	0.997		
Diabetes mellitus	24 (34.3)	123 (28.9)	1.29 (0.75–2.20)	0.409		
Renal disease	16 (22.9)	77 (18.1)	1.34 (0.73–2.47)	0.343		
Any malignancy	12 (17.1)	41 (9.6)	1.94 (0.97–3.91)	0.063		
Invasive pneumococcal disease	24 (34.3)	68 (16.0)	2.75 (1.57–4.80)	< 0.001	3.19 (1.55–6.59)	0.002
*Outcomes*						
ICU admission	31 (44.3)	23 (5.4)	13.93 (7.40–26.19)	< 0.001	23.22 (11.07–48.70)	< 0.001
Length of stay (days), median (IQR)	7 (4–21)	8 (6–15)		0.081		
Length of stay > 7 days	33 (47.1)	242 (56.8)	1.08 (0.90–1.29)	0.414		

Variables included in the final multivariable analysis were age group, gender, Charlson’s score > 3, diagnosis of invasive pneumococcal disease, and intensive care unit admission. *OR* odds ratio, *CI* confidence interval, *AOR* adjusted OR, *SD* standard deviation, *IQR* inter-quartile range, *ICU* intensive care unit

## Discussion

Among our cases comprising hospitalised patients with pneumococcal disease, majority were aged ≥65 years and of male gender, and about a quarter had a Charlson’s score of > 3. The ethnic distribution of the patients was reflective of the national census [22]. Compared to the control group, IPD patients demonstrated a trend towards being younger, and with lower Charlson’s scores. Non-IPD patients were more similar to the control group, but with only slightly lower mean age and Charlson’s scores. Patients in older age groups or with more co-morbidities might have had increased prior healthcare exposures, or had specific risk factors (such as swallowing impairment) which resulted in them being more likely to develop infections [23, 24] from other aetiologies other than *S. pneumoniae* [25, 26]. However, in contrast to the age and co-morbidity trends, the ICU admission and in-hospital mortality rates were highest in IPD patients and lowest in the control group. This reflects the virulence of the disease and highlights the importance of early recognition, treatment, and prevention where possible.

Our findings showed that prior pneumococcal vaccination was associated with a reduced risk of IPD by about 80% in our study population, after adjustments for age, gender, Charlson’s score and influenza vaccination status. This was consistent with studies in other settings evaluating the effectiveness of PCV13 and PPSV23 [13–15]. Pneumococcal serotypes covered by these vaccines have been shown to be well matched to those circulating in the Singapore population [6, 10, 27]. In particular, serotypes 3, 6B, 7F, 8, 19A and 23F are predominant in the 19-64 years age group, while serotypes 3, 14 and 19A are predominant among those 65 years and above [27].

In addition, 50 (54.4%) of the IPD cases among our hospital patients were below 65 years of age, of whom 24 (48.0%) had some form of chronic disease. A Japanese study of 10.4 million individuals demonstrated that younger adults with at least one medical condition were at greater risk of IPD compared to healthy older adults. For example, an adult aged 50-64 years with an underlying medical condition had a higher risk compared to a healthy adult aged ≥65 years [28]. Although the overall incidence in younger adults is lower than in older age groups [4, 29], the increased susceptibility of adults < 65 years with chronic diseases suggests that targeted efforts to vaccinate this population might be beneficial in reducing IPD incidence.

Currently, our hospital has ongoing vaccination programmes to identify and opportunistically vaccinate high-risk groups according to standardised protocols. In the inpatient setting, high-risk inpatients are counselled on and given vaccination prior to discharge, while in the outpatient setting, selected specialist clinics have a nurse-led programme for counselling and vaccination administration for high-risk patients, in conjunction with the medical consultation. Vaccination should also be provided by primary care practitioners as part of chronic disease management and alongside other preventive services such as health screening.

Our study, however, did not demonstrate any significant effect of PCV13 vaccination on reducing risk of pneumococcal disease, likely due to the low proportion of patients in our study with a clearly documented history of PCV13 vaccination. Apart from adult vaccination, widespread introduction of pneumococcal conjugate vaccine in children has been shown to reduce the incidence of IPD across all ages groups due to herd immunity, despite evidence of serotype replacement [30–33]. Singapore introduced PCV7 into its National Childhood Immunisation Programme in October 2009, and this was switched to PCV13 in December 2011. The cost-effectiveness of childhood vaccination in the Singapore setting has been demonstrated by Tyo et al. [34]. Future studies are warranted to study the effectiveness of increasing PCV13 vaccination uptake, in both adults and children, to reduce incidence of invasive and non-invasive pneumococcal disease.

Finally, our study also demonstrated key factors associated with in-hospital mortality among pneumococcal patients. ICU admission was the strongest predictor, with those having admission being 23 times as likely as those who did not to die during their admission. Other factors included age ≥ 85 years, higher Charlson’s score and diagnosis of IPD. Medical teams should especially note the increased mortality risk in these patients and ensure prompt management to reduce the risk of death.

The strengths of our study include a systematic selection of cases and controls among hospitalised patients. Furthermore, we followed up the patients longitudinally to the point of discharge from the hospital. Our study population is likely representative of patients hospitalised for pneumococcal disease in Singapore, as our hospital has a large catchment area which is not geographically restricted and patients from anywhere in Singapore may be admitted. Our findings are useful to guide the identification of patients at high risk of in-hospital mortality from the disease. They also support current recommendations to vaccinate at-risk individuals to prevent IPD.

Our study also has some limitations. Selection bias might have been present as clinicians may have preferentially ordered streptococcal urinary antigen and sterile site cultures for certain groups of patients, such as those who were elderly, with co-morbidities, or without records of recent vaccinations. At the time of the study, we had verified that there were no clinical protocols mandating such tests to be done for any particular patient groups. Our definition of cases included the use of results from streptococcal urinary antigen testing, which has an estimated sensitivity of 74.0% and specificity of 97.2% [35]. Sterile site cultures from multiple possible sources (e.g. blood, joint fluid, or pleural fluid) will also have varying microbial culture methods with different sensitivity and specificity. Some cases of pneumococcal disease may hence have been misclassified as controls (i.e. false negatives). However, such a misclassification could have reduced the magnitude of our findings, but not negated them. As our data was obtained through hospital electronic medical records, clinical data from other sources (e.g. other hospitals or the primary care sector) were not available. However, majority of our patients tended to return back to our hospital if future admissions were required. Moreover, it is likely that this issue would result in non-differential misclassification (if any) as data would not be captured differently across study groups. We were unable to capture data for some known risk factors, such as smoking history and socioeconomic status. Our analysis of IPD cases was limited by the relatively small numbers, especially when divided into subgroups. Pneumococcal serotype data were also not available for this study, as this is not routinely performed for isolates. Nevertheless, we have reviewed data from serotyping studies conducted in the local setting [6, 10, 27], which we believe to be applicable to our study population. The generalisability of our study is limited to hospitalised adult patients.

## Conclusion

In conclusion, while patients with pneumococcal disease tended to be younger and with less co-morbidities compared to those without pneumococcal disease, risk of ICU admission and death were nevertheless higher, especially in IPD patients. Mortality risk was highest in those with ICU admission, age ≥ 85 years, higher Charlson’s score and diagnosis of IPD. Pneumococcal vaccination effectiveness against IPD was estimated to be about 80%, and should be encouraged among high-risk patients, including those from younger age groups.

## Data Availability

The datasets used and/or analysed during the current study are available from the corresponding author on reasonable request.

## Abbreviations

IPD: Invasive pneumococcal disease
ICU: Intensive care unit
CAP: Community-acquired pneumonia
PCV13: 13-valent pneumococcal conjugate vaccine
PPSV23: 23-valent pneumococcal polysaccharide vaccine
ACIP: Advisory Committee on Immunisation Practices
MOH: Ministry of Health
TTSH: Tan Tock Seng Hospital
ICD-10: International Classification of Diseases, 10th Revision
IQR: Interquartile range
RRR: Relative risk ratio
OR: Odds ratio
CI: Confidence interval
AOR: Adjusted odds ratio
